# Sulfur Interaction and Regeneration of CaMn_0.775_Ti_0.125_Mg_0.1_O_2.9−δ_ Perovskite as Oxygen Carrier during Combustion of Sour Gas in a 500 W_th_ Chemical Looping Combustion Unit

**DOI:** 10.1021/acs.energyfuels.3c02391

**Published:** 2023-08-29

**Authors:** A. Cabello, A. Abad, T. Mendiara, M. de las Obras Loscertales, L. F. de Diego

**Affiliations:** † Department of Energy and Environment, Instituto de Carboquímica (ICB-CSIC), Miguel Luesma Castán 4, Zaragoza 50018, Spain

## Abstract

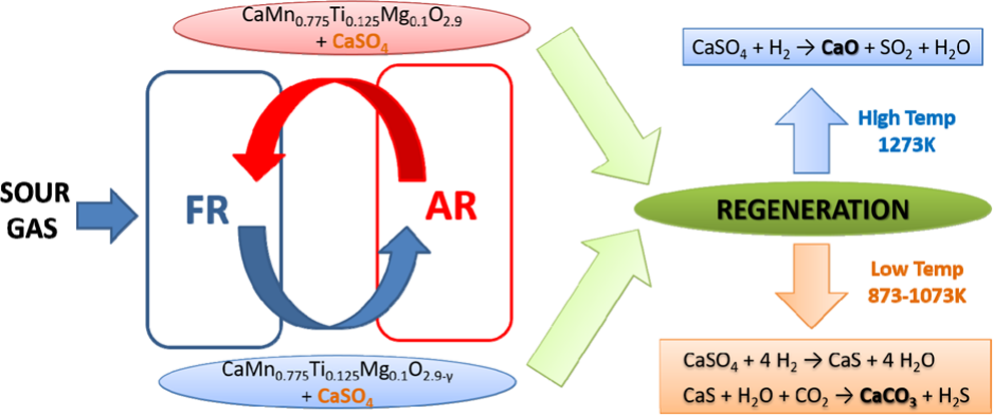

In the present study, the performance of a CaMn_0.775_Ti_0.125_Mg_0.1_O_2.9−δ_ perovskite used as an oxygen carrier to burn sour gas with different H_2_S concentrations (up to 3000 vppm) in a continuous 500 W_th_ chemical looping combustion (CLC) prototype was investigated. After 29 h of sour gas combustion, the combustion efficiency had dropped by 18% in comparison with the reference test without sulfur addition. The characterization of the used particles of the perovskite confirmed that the presence of sulfur in the fuel gas had a poisonous effect through the formation of undesired compounds, such as CaSO_4_. The reactivity with CH_4_ and oxygen uncoupling capacity decreased, which could explain the decrease in the combustion efficiency. Two regeneration processes, one at high temperature (1273 K) and another one at low temperature (773–873 K), were carried out in a batch fluidized bed reactor to remove the amount of sulfur accumulated in the oxygen carrier particles. The detection of appreciable amounts of gaseous sulfur-based compounds (SO_2_ and H_2_S) during the experimentation and the postcharacterization results obtained through different techniques such as X-ray diffraction, ultimate analysis, and thermogravimetric analysis confirmed the effectiveness of both processes. Finally, the feasibility of implementation of the regeneration processes in a commercial CLC unit was thoroughly analyzed.

## 1 Introduction

Contemporary society is increasingly aware of the reality of climate change and the need to reduce greenhouse gas (GHG) emissions from the atmosphere in order to mitigate this worldwide threat. The Paris Agreement within the United Nations Framework Convention on Climate Change (UNFCCC) seeks to prevent the global average temperature from raising more than 2 °C by the end of this century.^1^ Carbon capture and storage (CCS) is, according to all predictions, one of the technologies with the highest potential to contribute to the achievement of this objective. In this regard, chemical looping combustion (CLC) is a very promising technology for CO_2_ capture from a techno-economic point of view, since it presents the least loss of energy efficiency compared to a facility without a CO_2_ capture system.^2^

CLC technology can be classified as a second-generation oxy-combustion technology since combustion takes place in the absence of N_2_. However, unlike oxy-combustion, the oxygen required for the combustion process is not obtained through an air separation unit (ASU), but indirectly by means of a solid oxygen carrier (usually a metal oxide of a transition element), making the CO_2_ capture inherent to the process. The oxygen carrier has to be able to continuously both supply oxygen to the fuel and capture oxygen from air at high temperatures (973–1273 K) to oxidize the fuel and be regenerated, respectively. Even though there are different configurations to perform a CLC process, the configuration composed of two interconnected fluidized bed reactors, known as fuel reactor (FR) and air reactor (AR), has been the most widely used up to date. Generically, the reactions that take place in both reactors are the following:
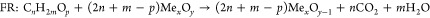
R.1
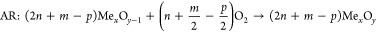
R.2

The sum of reactions R.1 and R.2 shows that the net chemical reaction, as well as the total enthalpy of combustion, is the same as that of conventional combustion with air; see reaction R.3.
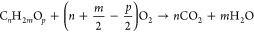
R.3

The material selected as oxygen carrier for a CLC process must meet two essential requirements: (1) high conversion selectivity of the fuel to CO_2_ and H_2_O and (2) high reactivity with fuel and air during thousands of reduction and oxidation cycles. Likewise, it is highly recommended that it is not prone to agglomeration issues, which would make its use in fluidized beds difficult, or carbon deposition, and it must exhibit high resistance to attrition. Other relevant aspects to take into account at the time of selecting a suitable oxygen carrier are its cost and benignity from an environmental point of view. In addition, its poisoning propensity should be considered when sulfurous fuels are used.

A great variety of oxygen carriers have been developed to date for the CLC process with solid, liquid, and gaseous fuels, with those based on copper, nickel, manganese, and iron oxide having the most suitable properties.^3−7^ Among them, the metal oxides based on copper and manganese present a very interesting feature that lies in their ability to uncouple oxygen in gaseous phase under the conditions existing in the fuel reactor.^8^ The process that uses this kind of material is known as chemical looping with oxygen uncoupling (CLOU), which promotes the conversion of a fuel by direct combustion with released O_2_. In the specific case of the combustion technology for gaseous fuels through the CLOU process, CaMnO_3_-based perovskite materials have aroused great interest within the scientific community thanks to their high reactivity with natural gas and suitable mechanical stability.^9−21^ Cabello et al.^22^ recently summarized all the experimental experience of CaMnO_3_-based perovskites in continuous CLC plants with gaseous fuels.

The amount of molecular oxygen released by this type of material is given by the following general reaction:
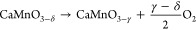
R.4where γ and δ are the parameters in the subscript for oxygen in the reduced and oxidized states, respectively.

The total oxygen transport capacity of this type of material is very high with a portion generated through the uncoupling reaction R.4. Another portion is transferred via lattice oxygen according to the following reaction—with methane as fuel as an example:
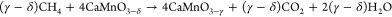
R.5

The large amount of calcium content in the structure of this type of perovskite makes them very sensitive to the presence of sulfur in the fuel fed into the CLC unit. Cabello et al.^23^ evaluated the behavior of a CaMg_0.1_Mn_0.9_O_3−δ_ perovskite in a continuous 500 W_th_ CLC plant when the sour gas used as fuel contained H_2_S concentrations of up to 3400 vppm. They observed immediate poisoning of the material through a sudden decrease in combustion efficiency and reactivity. After 17 h of combustion with addition of sulfur, the combustion efficiency had dropped by 27%, and the operation had to be interrupted due to evident signs of agglomeration in the oxygen carrier particles. In terms of reactivity, the presence of sulfur in the fuel gas affected both the reaction rate between CH_4_ and the CaMg_0.1_Mn_0.9_O_3−δ_ oxygen carrier, as well as the oxygen uncoupling capability of the perovskite with significant decreases in both parameters. In this regard, it is important to note that the oxygen uncoupling capability in this type of material is very relevant to obtain high values of natural gas combustion efficiency.^24^

Pachler et al.^25^ analyzed the performance of a CaMn_0.775_Mg_0.1_Ti_0.125_O_3−δ_ perovskite in a larger continuous CLC plant (120 kW_th_) during the combustion of natural gas with H_2_S concentrations of up to 3000 vppm. These researchers also observed that the presence of sulfur was detrimental, as fuel conversion slowly but steadily declined throughout the experimental campaign. However, they did not evaluate how the presence of this compound affected the reactivity of the particles and whether sulfur deactivation had more influence on the reaction rate with CH_4_ or on the oxygen uncoupling capability of the perovskite. Furthermore, sulfur was accumulated on the surface of the oxygen carrier particles as MgS, but it was not quantified, and the material was not capable of regenerating under CLC conditions when the H_2_S feed was shut off.

Despite the evident influence of the presence of sulfur on the behavior of this type of material in CLC processes, there is a significant lack of literature on regeneration processes for oxygen carriers based on CaMnO_3_ perovskites when they react with gaseous fuels that contain sulfur. Only a few solutions have been proposed which are based on carrying out a deep reduction with H_2_ in a thermogravimetric analyzer (TGA)^26^ or heating in absence of sulfur in order to remove the accumulated sulfur in form of CaSO_4_^27^ or MgS.^25^

In this work, the performance of a CaMn_0.775_Ti_0.125_Mg_0.1_O_2.9−δ_ perovskite as an oxygen carrier to burn sour gas with different H_2_S concentrations was examined in a continuous 500 W_th_ CLC prototype. A thorough characterization of the solids particles was performed to better understand the effects of the presence of H_2_S on CH_4_ reactivity, oxygen uncoupling capacity, and mechanical strength for this kind of perovskite, as well as to quantify the amount of sulfur accumulated in the material and the distribution of this poisonous compound in the particles. In addition, two regeneration processes were implemented: (1) redox cycles, including reduction with H_2_ and oxidation in air at high temperature (1273 K) and (2) H_2_S formation with CO_2_ and H_2_O mixtures at low temperature (<873 K). Finally, the possibilities of implementing these regeneration processes in a commercial CLC unit were discussed.

## 2 Experimental Section

### 2.1 Oxygen Carrier

The CaMnO_3−δ_-based perovskite used as an oxygen carrier in this work, whose chemical formula is CaMn_0.775_Ti_0.125_Mg_0.1_O_2.9−δ_, was prepared by VITO following the spray drying method. The oxygen carrier production was scaled up using industrially available raw materials throughout the EU funded project SUCCESS.^28^ The material, named C28, was manufactured using the following raw commercial materials: Ca(OH)_2_ from KÖ-SL (Nordkalk), Mn_3_O_4_ from Colormax P (Elkem), TiO_2_ from M211 (Sachtleben), and MgO from Magchem 30. The spray-dried particles were calcined at 1608 K for 4 h. Further details about the preparation method can be found elsewhere.^18^Table 1 shows the main physicochemical characteristics of the freshly received sample and of the used particles at the end of the experimental campaign in the continuous CLC prototype with H_2_S addition. Regarding XRD characterization, only CaMnO_3_ and MgO were detected as crystalline phases in the fresh particles. At this point, it is worth mentioning that the presence of Ti in the perovskite structure cannot be distinguished from the CaMnO_3_ phase by XRD.

**Table 1:** Main Characteristics of Fresh and Used C28 Oxygen Carrier Particles

	Fresh particles	Used particles (Test 6, 50 h, AR)
Oxygen transport capacity, *R*_OC,t_ (%)	8.5	8.0
Mean particle size (μm)	171	160
Specific surface area, BET (m^2^/g)	0.28	0.31
Porosity (%)	36.7	34.2
Mechanical strength (N)	1.6	1.5
XRD crystalline phases	CaMnO_3_, MgO	CaMnO_3_, MgO, CaTiO_3_, CaSO_4_

### 2.2 Oxygen Carrier Characterization Techniques

The main physicochemical properties of the oxygen carrier particles were determined by a series of characterization techniques. The oxygen transport capacity is defined as the mass fraction of oxygen present in the oxygen carrier that can react with a gaseous fuel. In the particular case of CaMnO_3−δ_-based perovskite materials, two different oxygen transport capacities can be determined:^21^

(1) The total oxygen transport capacity, *R*_OC,t_, which is defined as the total fraction of oxygen that can react with the fuel.
(2) The oxygen transport capacity for the oxygen uncoupling, *R*_OC,ou_, which is defined as the oxygen available in the particulate material for the oxygen uncoupling reaction and primarily depends on the reaction temperature.^16^

The mean particle size of the perovskite was determined through a laser diffraction technique according to the ISO 13320 standard in LS 13320 Beckham Coulter equipment. The specific surface area was calculated by the Brunauer–Emmett–Teller (BET) method using N_2_ as adsorbate at 77 K in Micromeritics ASAP-2020 equipment. Total pore volume and pore size distribution were determined by mercury porosimetry in an AUTOPORE V apparatus. The crushing strength of the oxygen carrier particles was measured by using a Shimpo FGN-5X dynamometer. The actual value of this parameter was taken from at least 20 measurements. The attrition resistance was determined using a three-hole air jet attrition tester, Model ATT-100M, configured according to ASTM-D-5757-95.^29^ As specified in the ASTM method, 50 g of material, in this case fresh and used samples after 50 h of operation, was tested under an air flow of 10 L/min, and the weight loss of fines was recorded at 1 and 5 h of time on stream, respectively. The percentage of fines after a 5-h test is the air jet attrition index (AJI). According to this ASTM method, particles smaller than 20 μm in size are considered as fines. XRD diffraction analyses were carried out in a Bruker D8 Advance A25 powder X-ray diffractometer equipped with an X-ray source with a Cu anode working at 40 kV and 40 mA and an energy-dispersive one-dimensional detector. The diffraction pattern was obtained over the 2θ range from 10° to 80° with a step of 0.019°. The assignation of crystalline phases was performed according to the Joint Committee on Powder Diffraction Standards. DIFFRAC.EVA software supports a reference pattern database derived from the Crystallography Open Database (COD) and the Powder Diffraction File (PDF) for phase identification. The microstructure and morphology of the oxygen carrier particles were analyzed by means of a scanning electron microscope SEM EDX Hitachi S-3400N equipped with an EDX analyzer Röntec XFlash of Si (Li). Finally, in order to quantify the possible presence of sulfur in the perovskite particles, some samples were analyzed by ultimate analysis in a Thermo Flash 1112.

### 2.3 500 W_th_ Continuous CLC Unit

**Figure 1:**
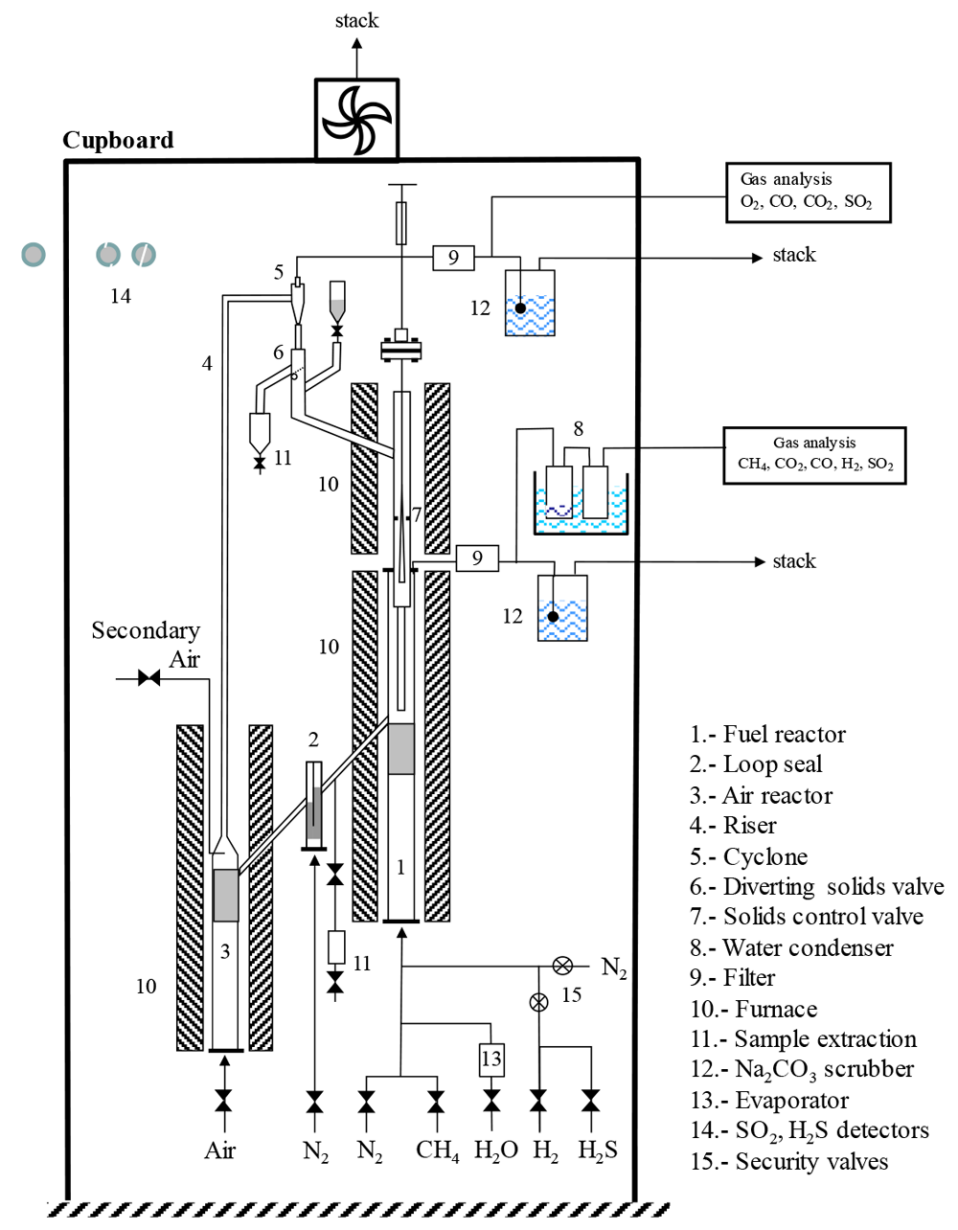
Schematic diagram of the ICB-CSIC-g1 prototype to operate with sour gas containing high concentrations of sulfur.

Figure 1 illustrates a scheme of the CLC prototype used to assess the performance of the oxygen carrier under continuous combustion of sour gas with different concentrations of H_2_S. This unit, named ICB-CSIC-g1, was modified to operate safely when sour gases with high concentrations of H_2_S are used. A detailed description of such modifications can be found elsewhere.^30^

The ICB-CSIC-g1 unit is composed of two interconnected bubbling fluidized bed reactors that operate at atmospheric pressure, namely, the fuel reactor (1) and the air reactor (3), a loop seal (2), a riser (4) to transfer the particulate material between reactors, a cyclone (5) to separate the solids particles from the vitiated air stream and return them to the fuel reactor, and two filters (9) to collect the particles escaping with gases during the continuous CLC process. An important feature of the ICB-CSIC-g1 prototype lies in the possibility of measuring and controlling the solid circulation rate at any time by means of a diverting solids valve (6) and a solids control valve (7). This CLC unit also allows for the collection of solid material samples from both reactors (11) at any time of the experimental campaign for further characterization. Furthermore, the prototype is provided with several tools to measure and control its main operating conditions such as temperature, pressure, and gas mass flows. Specifically, thermocouples, pressure transducers, and mass flow controllers are located at different points of the plant. The ICB-CSIC-g1 facility also has online gas analyzers to get continuous data of gas composition at the fuel reactor and air reactor outlet streams. CO, CO_2_, CH_4_, and SO_2_ dry basis concentrations are determined using nondispersive infrared analysis (NDIR), and H_2_ concentration is measured by thermal gas conductivity. The O_2_ concentration is determined in a paramagnetic analyzer.

### 2.4 Testing Conditions in the Continuous CLC Unit

The experimental campaign in the continuous bench-scale CLC plant lasted 50 h, from which 29 h were at combustion conditions using sour gas as fuel with H_2_S concentrations up to 3000 vppm. The temperatures in the fuel reactor and air reactor were fixed throughout the whole experimental campaign at 1173 and 1223 K, respectively. The gas flow fed to both reactors was identical for all the sour gas combustion tests. The air reactor was fluidized with air, and the gas flow was divided into the primary air, added from the bottom bed (720 L_N_/h), and the secondary air, added at the top of the bubbling bed to help particle entrainment in the riser (150 L_N_/h). The fluidization velocity in the air reactor was 0.44 m/s, whereas this parameter reached a value of 2.5 m/s in the riser. In the case of the fuel reactor, the inlet gas flow was fixed at 192 L_N_/h, corresponding to a gas velocity of 0.11 m/s. The gas stream fed to the fuel reactor was composed of CH_4_ (15 vol %), H_2_ (5 vol %), H_2_O (10 vol %), H_2_S (0–3000 vppm), and N_2_ to balance. Hydrogen was added to avoid H_2_S decomposition in the feeding line before entering the reactor, and steam was fed to avoid corrosion issues with reactor alloys. This flow and gas composition resulted in an input power to the CLC unit of around 315 W which, considering the amount of material present in the fuel reactor throughout the experimental campaign (260 g approximately), signified a solids inventory of 825 kg/MW. Finally, pure N_2_ was also used to fluidize the bottom loop seal (37.5 L_N_/h). Table 2 shows a summary of the main variables used throughout the sour gas combustion tests carried out with the C28 material in the ICB-CSIC-g1 prototype.

**Table 2:** Sour Gas Combustion Tests with a C28 Oxygen Carrier

Test	H_2_S in (vppm)	*ṁ*_*s*_ (kg/h)	ϕ (−)	Accumulated time with H_2_S (h)
1	0	19.0	19.7	0
2	100	18.4	19.1	2
3	500	18.4	19.1	2.8
4	1000	18.4	19.1	3.5
5a	3000	18.4	19.1	8
5b	3000	18.4	19.1	15.5
5c	3000	18.1	18.8	23
5d	3000	12.5	13.0	25
6	100	12.5	13.0	29

Test 1 was performed without the presence of H_2_S in the fuel gas in order to establish it as a reference test considering the possible deactivation of the perovskite when this material reacts with the previously mentioned gaseous mixture. This test was carried out with a high oxygen carrier-to-fuel ratio (ϕ = 19.7) in order to maximize the combustion efficiency at the beginning of the experimental campaign as shown in a previous work with this perovskite.^22^ The oxygen carrier-to-fuel ratio (ϕ) is defined as the ratio between the oxygen available in the oxygen carrier (assuming completely oxidized in the air reactor) and the oxygen needed to stoichiometrically achieve the complete combustion of the fuel. A value of 1 corresponds to the stoichiometric oxygen carrier circulation needed for complete conversion of the gas to CO_2_, SO_2_, and H_2_O, calculated by eq E.1 as follows:
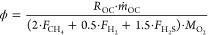
E.1where *R*_OC_ is the oxygen transport capacity of the oxygen carrier, *m*_OC_ is the solids circulation flow rate (kg/s), *F*_*i*_ is the molar flow of component *i* (mol/s), and *M*_O_2__ is the molecular weight of gaseous oxygen (kg/mol).

The effect of the presence of H_2_S in the feeding gas was evaluated in tests series 2–5. Four different H_2_S concentrations in the fuel gas were considered, ranging from 100 to 3000 vppm. Finally, in test 6, the concentration of H_2_S was lowered again to 100 vppm, and it was analyzed whether the results from test 2 could be achieved, or on the contrary, the long operating time with a high concentration of H_2_S in the fuel gas had a permanent detrimental effect on the behavior of the oxygen carrier. In all the cases, the continuous CLC pilot plant was operated with a high excess of oxygen carrier circulation (ϕ = 13.0–19.1). Considering the unit design and the solid circulation rate used, the CaMn_0.775_Ti_0.125_Mg_0.1_O_2.9−δ_ perovskite particles were subjected to about 355 redox cycles in the ICB-CSIC-g1 facility.

The performance of the C28 material in the 500 W_th_ CLC unit was evaluated by means of the combustion efficiency parameter, η_c_. It is defined as the ratio of oxygen consumed by the gas leaving the fuel reactor to that consumed by the gas when the fuel is completely burned to form CO_2_, H_2_O, and SO_2_. A η_c_ value close to 1 indicates that the CLC plant achieves full combustion of the supplied fuel during operation.
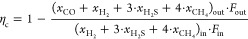
E.2where *F*_in_ and *F*_out_ are the molar flows at the inlet and outlet of the fuel reactor, respectively, and *x*_*i*_ is the molar fraction of gas *i*.

### 2.5 Batch Fluidized Bed

**Figure 2:**
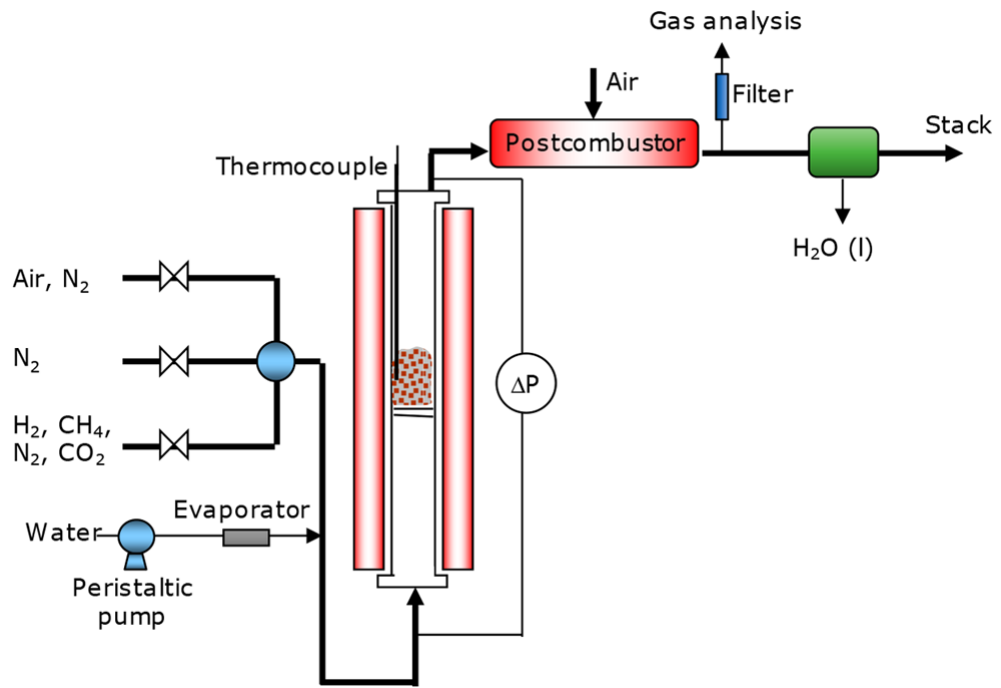
Schematic layout of the batch fluidized bed reactor.

The regeneration processes to eliminate the amount of sulfur accumulated in the particles of the C28 material were carried out in a batch fluidized bed reactor similar to the one shown in the scheme of Figure 2. The reactor, 55 mm I.D., and 830 mm height, is electrically heated by means of a furnace and has a preheating zone under the distributor plate. The facility has a thermocouple and pressure taps to measure the temperature inside the bed of oxygen carrier particles and the pressure drop, respectively. The possible presence of agglomeration or defluidization problems can be detected by a sharp decrease in the bed pressure during operation. The fluidized bed reactor can be fed with different gases during the reduction (CH_4_, H_2_, CO_2_, N_2_), purge (N_2_), and oxidation (air and N_2_) stages. In addition, the experimental setup had a peristaltic pump and an evaporator to feed steam.

Two different procedures have been tested for oxygen carrier regeneration:

(1) The high-temperature regeneration tests were conducted at 1273 K using H_2_/N_2_ mixtures (10 vol % H_2_/90 vol % N_2_) and air/N_2_ mixtures (10 vol % O_2_/90 vol % N_2_) during the reduction and oxidation stages, respectively.
(2) The regeneration tests by H_2_S formation with CO_2_/H_2_O mixtures at low temperature were performed according to the following methodology: first, the reactor was heated in a N_2_ atmosphere up to 1073 K. Then, at this temperature, the oxygen carrier was reduced with H_2_ (15 vol % H_2_/85 vol % N_2_). Once the CaMn_0.775_Ti_0.125_Mg_0.1_O_2.9−δ_, particles were completely reduced, the temperature was decreased to 773 K in a N_2_ atmosphere. Finally, a mixture of CO_2_ and steam (50 vol % CO_2_/50 vol % H_2_O) was fed into the reactor to begin regeneration. Temperature and gas composition were selected as the optimum to regenerate CaS by this process.^31^ A postcombustor is installed at the reactor outlet to oxidize any unburned compounds. In this work, a 200 L_N_/h air stream was fed into the postcombustor to oxidize the H_2_S formed during the regeneration processes carried out at low temperature to SO_2_. Finally, for all the regeneration tests, the solids inventory in the reactor and the fluidization gas velocity were 250 g and 0.12 m/s, respectively. The gas analysis system was similar to that described for the CLC unit.

## 3 Results

### 3.1 Effect of H_2_S Concentration on the Fuel Combustion in the CLC Facility

A batch of 1.5 kg of CaMn_0.775_Ti_0.125_Mg_0.1_O_2.9−δ_ perovskite particles was used to perform a series of combustion tests in the continuous 500 W_th_ CLC facility. The main goal of these tests was to evaluate the effect of the presence of H_2_S in the fuel gas in terms of combustion efficiency, gas product distribution, sulfur splitting between reactors, oxygen carrier reactivity, and agglomeration. In addition, these tests allowed for obtaining used samples under real operating conditions and in the required quantities to conduct both a comprehensive physicochemical characterization and the corresponding regeneration tests.

**Figure 3:**
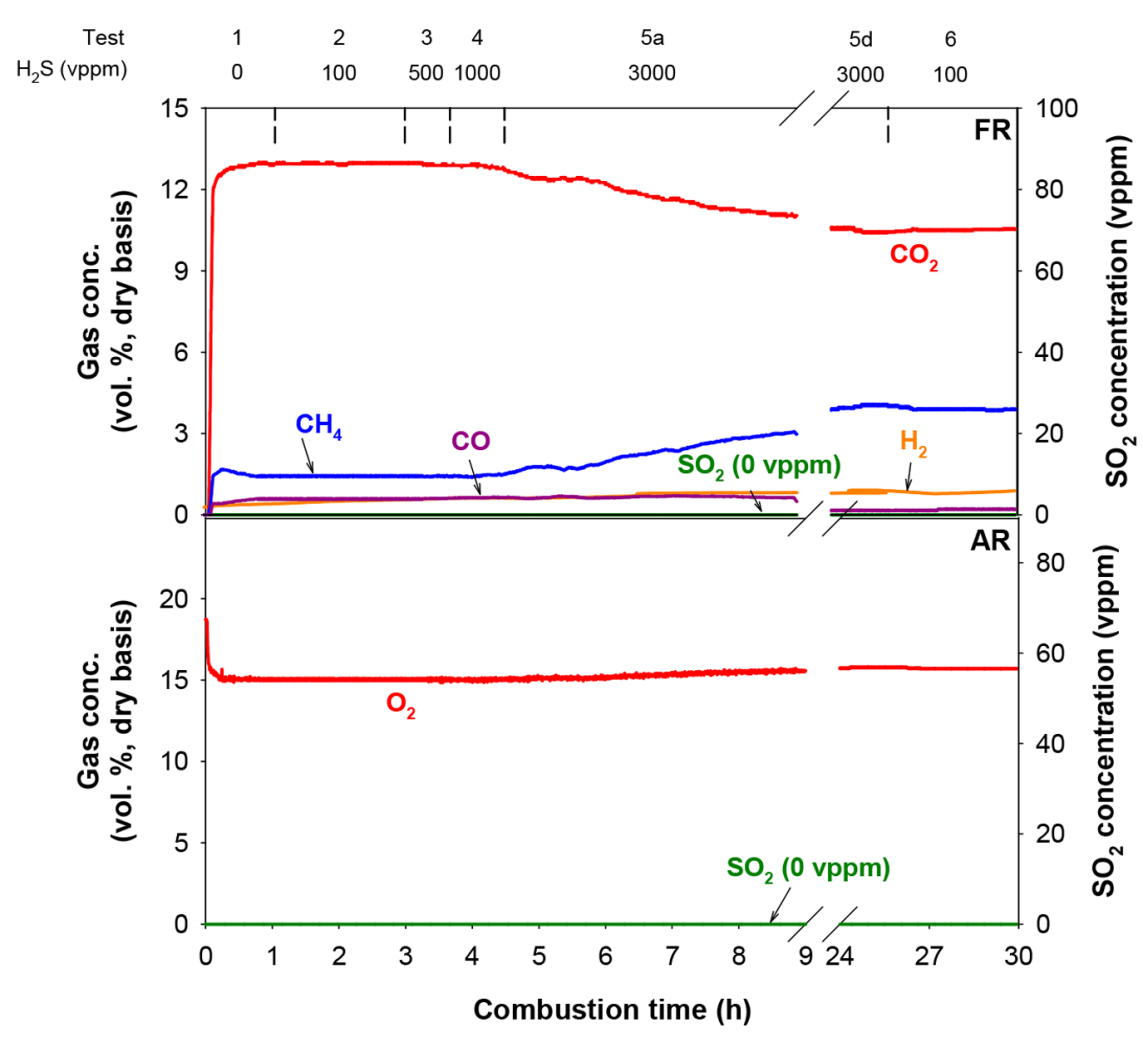
Gas product distribution obtained at the outlet of fuel and air reactors corresponding to tests 1–6 with the CaMn_0.775_Ti_0.125_Mg_0.1_O_2.9−δ_ perovskite.

Figure 3 shows the gas product distribution obtained at the outlet of fuel reactor and air reactor during tests 1–6. Test 1 was carried out without sulfur addition and with a high excess of lattice oxygen available in the fuel reactor (ϕ = 19.7). The concentrations of CO_2_ and CH_4_ leaving the fuel reactor were stabilized at 13 and 1.4 vol %, respectively. Regarding H_2_ and CO concentrations, they were very similar to values lower than 0.5 vol %. Tests 2–4 were performed at similar conditions to test 1 in terms of oxygen carrier-to-fuel ratio (ϕ = 19.1), but with sulfur addition. Under these conditions, similar CO_2_, CH_4_, H_2_, and CO concentrations at the outlet stream of the fuel reactor were measured. However, when the H_2_S concentration was increased up to 3000 vppm in test 5a, maintaining constant the oxygen carrier-to-fuel ratio parameter (Table 2), CH_4_ concentration slowly increased, indicating a decrease in the reactivity of the oxygen carrier particles. Significant variations in the oxygen carrier performance were observed 4.5 h later at the end of the test, and no stationary state conditions were reached yet. During test 5d, after more than 20 h of operation with addition of 3000 vppm of H_2_S, the concentrations of CO_2_ and unburned CH_4_ at the outlet of the fuel reactor were stabilized at 10.5 and 3.9 vol %, respectively. Finally, during test 6, the H_2_S concentration was reduced to 100 vppm, and it was found that the results of test 2 could not be replicated with the same concentration of H_2_S since the gas product distribution at the outlet of the fuel reactor was almost identical to that of the previous experiment with high H_2_S concentration (test 5d). This result revealed that the addition of small amounts of H_2_S together with the fuel gas had a cumulative effect on the behavior of the oxygen carrier in terms of the combustion efficiency. Furthermore, it must be pointed out that there was no detection of SO_2_ at the outlet stream of both reactors at any time during the experimental campaign, evidencing that all of the sulfur present in the fuel gas was being accumulated in the oxygen carrier particles.

This behavior of the CaMn_0.775_Ti_0.125_Mg_0.1_O_2.9−δ_ perovskite is different from that exhibited during an experimental campaign conducted in a 120 kW_th_ chemical looping combustion pilot unit^25^ with similar H_2_S concentrations (100–3000 vppm) in which a considerable fraction of sulfur was detected as SO_2_ at the outlet of the fuel reactor. The main difference between both experimental campaigns that could explain this disagreement is the operating temperature of the fuel reactor. While Pachler et al.^25^ operated the reactor at 1226 K, the temperature was limited to 1173 K in this work. On the contrary, particles of another CaMnO_3_-based perovskite with formula CaMn_0.9_Mg_0.1_O_3−δ_, denominated as C14, were subjected to CH_4_ combustion tests with the presence of H_2_S in the ICB-CSIC-g1 prototype showing a performance very similar to that obtained in this work. In that case, only 3% of the total sulfur fed with the fuel was released as SO_2_ at the outlet of the fuel (0.5%) and air (2.5%) reactors. The remaining sulfur was gradually accumulated in the particles of the CaMn_0.9_Mg_0.1_O_3−δ_ perovskite.^23^

Finally, it is also noteworthy that neither CO_2_ nor CO was detected in the air reactor gas stream, which indicated the absence of gas leakage between reactors and carbon formation on the oxygen carrier particles in the fuel reactor.

**Figure 4:**
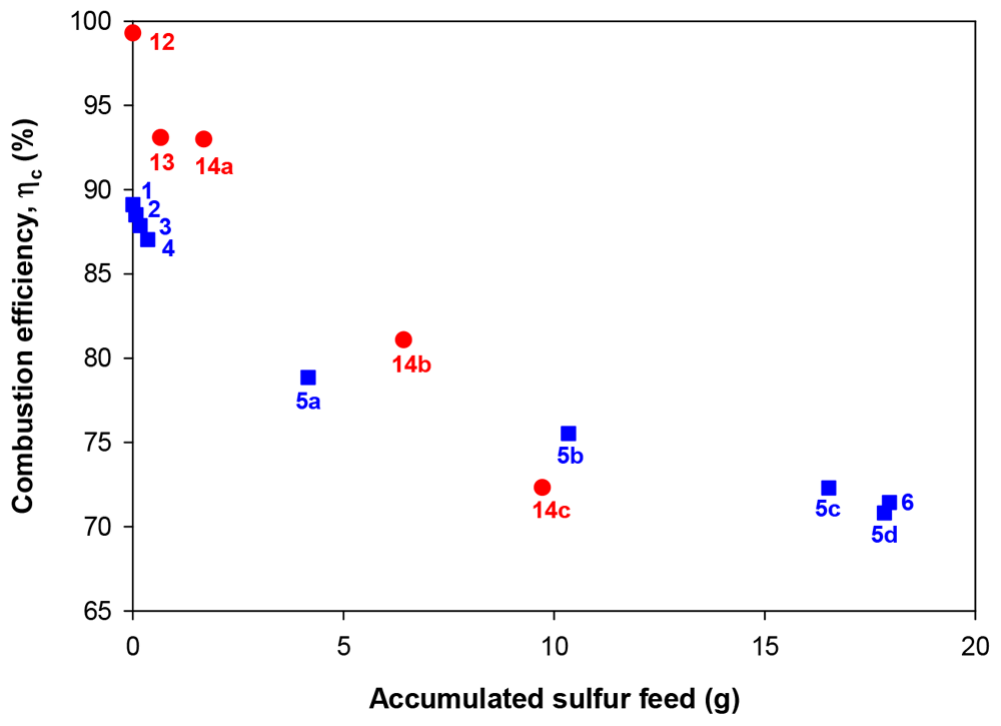
Effect of the amount of sulfur fed in the fuel reactor on the combustion efficiency (η_c_) for sour gas combustion with the C28 perovskite material (blue squares). Numbers correspond to the tests given in Table 2. *T*_FR_ = 1173 K; *T*_AR_ = 1223 K. Sour gas combustion results with the CaMn_0.9_Mg_0.1_O_3−δ_ material (red circles) in the same CLC prototype were also added for comparison purposes.^23^

Figure 4 shows the effect of the amount of sulfur fed into the fuel reactor on the combustion efficiency for each test performed within this experimental campaign (blue squares). The tests conducted in the same CLC unit with addition of H_2_S using a CaMn_0.9_Mg_0.1_O_3−δ_ perovskite as oxygen carrier have been also included for comparison purposes (red circles).^23^ The combustion efficiency during test 1, without sulfur addition, was 89%, which implied that even operating with a very high excess of oxygen carrier circulation, i.e., ϕ = 19.7, the CaMn_0.775_Ti_0.125_Mg_0.1_O_2.9−δ_ perovskite could not completely convert CH_4_ to CO_2_ and H_2_O. Continuous combustion experiments in this CLC plant concluded that it was necessary to operate at even higher oxygen carrier-to-fuel ratios to reach full combustion conditions with CaMnO_3_-based perovskite materials.^22^ At this point, it is worth mentioning that initial operating conditions were chosen in such a way that full CH_4_ combustion was not achieved in order to better assess the possible deactivation effect of the oxygen carrier by the addition of H_2_S.

The addition of sulfur did not cause a sharp drop in the combustion efficiency value, as occurred with the CaMn_0.9_Mg_0.1_O_3−δ_ material. In this work, during the first 3.5 h of sour gas combustion with H_2_S concentrations between 100 and 1000 vppm (tests 2–4), the combustion efficiency only decreased by 2% maintaining the ϕ parameter roughly constant. However, Cabello et al.^23^ reported a drop of almost 6% during the first 5 h of combustion with a H_2_S concentration of 450 vppm, corresponding to a sulfur feed of only 0.6 g, using the CaMn_0.9_Mg_0.1_O_3−δ_ material. This different behavior seems to indicate a greater capacity of sulfur accumulation without poisoning effects by the CaMn_0.775_Ti_0.125_Mg_0.1_O_2.9−δ_ material, at least with low sulfur concentration in the oxygen carrier particles.

In test 5a, the H_2_S concentration in the fuel gas was increased up to 3000 vppm, which brought about an important decrease of the combustion efficiency (η_c_ = 78%). Nevertheless, this sulfur concentration was maintained in the sour gas stream fed to the fuel reactor during 17 h more (tests 5b–5d) inducing a slow and gradual decrease of the combustion efficiency. After 25 h of sour gas combustion, with an addition of almost 18 g of sulfur to the CLC prototype, the η_c_ parameter reached a value of 71%, which supposed a drop of 18 percentage points in comparison to the reference test (test 1). However, it should be noted that the excess of oxygen in the reference test was higher than in test 5d with ϕ values of 19.7 and 13.0, respectively. In contrast, the CaMn_0.9_Mg_0.1_O_3−δ_ perovskite exhibited a higher sensitivity to sulfur poisoning with a decrease in combustion efficiency of 27% and only half of the sulfur fed (9.7 g) throughout 17 h of operation with H_2_S addition.^23^

Finally, a test with a very low H_2_S concentration (100 vppm) was repeated at the end of the experimental campaign (test 6) to differentiate the effect of the sulfur concentration from the time of operation on the combustion efficiency. In this regard, it was found that the accumulation of sulfur in the oxygen carrier particles had a more important effect on the reactivity than the H_2_S concentration itself, since the combustion efficiency in test 6 (100 vppm) was similar to the one achieved in test 5d (3000 vppm) at similar operating conditions (ϕ = 13.0).

### 3.2 Oxygen Carrier Characterization

In order to determine the effects of the presence of H_2_S in the fuel gas on the behavior of the CaMn_0.775_Ti_0.125_Mg_0.1_O_2.9−δ_ oxygen carrier particles, several samples were taken from the air and fuel reactors throughout the experimental campaign and were subjected to different characterization techniques.

**Figure 5:**
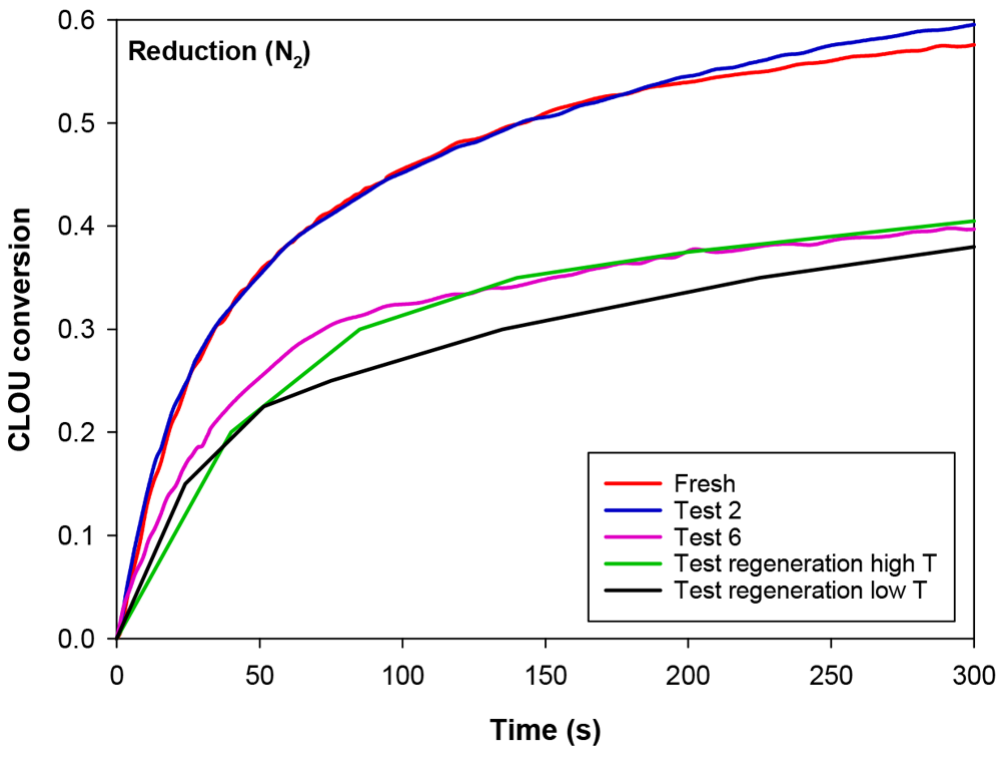
Oxygen uncoupling conversion of the C28 material at different times of operation in the CLC unit and after regeneration tests at low and high temperature. *T* = 1173 K.

The oxygen transport capacity and reactivity of the oxygen carrier particles were determined in a TGA CI Electronics type, following the procedure described elsewhere,^32^ both for the oxygen uncoupling reaction and gas–solid reaction with a reducing gas. Figure 5 shows the conversion vs time curves obtained for the oxygen uncoupling capability. The oxygen uncoupling conversion values were calculated assuming that the capacity to release oxygen of this perovskite was *R*_OC,ou_ = 1.4 wt % at 1173 K,^32^ the temperature at which TGA tests were conducted. At the beginning, the oxygen uncoupling reaction was relatively fast and then progressively slowed down over time. The oxygen uncoupling conversion achieved a value of 0.6 after 300 s in N_2_ for the fresh particles. This parameter barely changed after the first hours of operation using sour gas as the fuel (test 2). However, at the end of the combustion tests with H_2_S, the oxygen carrier particles had undergone a substantial decrease on their reactivity. Specifically, the initial reaction rate decreased, and the conversion achieved after 300 s in N_2_ was 0.4 for the particles extracted from the CLC unit at the end of test 6.

**Figure 6:**
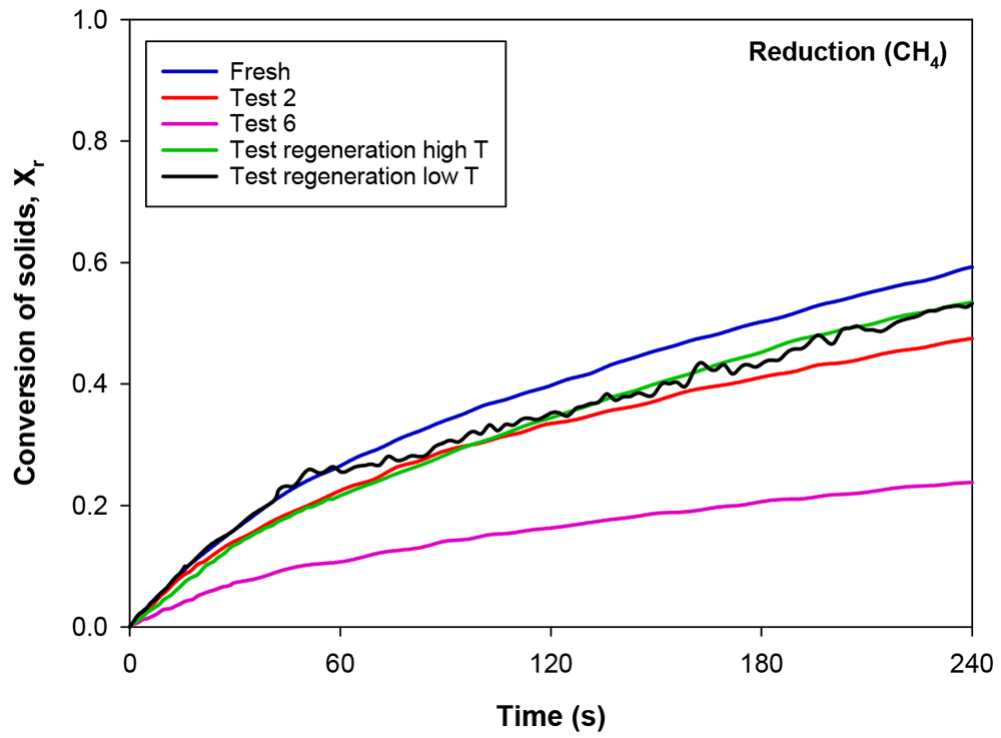
Reduction conversion versus time curves for the C28 material at different times of operation in the CLC unit and after regeneration tests at low and high temperatures. *T* = 1173 K.

The total oxygen transport capacity of the perovskite material with CH_4_ as reducing gas was *R*_OC,t_ = 8.5 wt %. This property was maintained almost constant throughout the time of operation in the CLC unit with a final value of 8.0 wt %; see Table 1. However, the reactivity of the material with CH_4_ determined through the TGA technique underwent a significant decrease, as can be clearly observed in Figure 6. After the first 2 h of sour gas combustion with sulfur addition, the reduction conversion (*X*_r_) of the particles decreased 10% approximately after 240 s of reaction. However, this deactivation was practically negligible in terms of combustion efficiency, see Figure 4, because all the experimental tests carried out in this work were conducted with a very high excess of oxygen (ϕ > 13), which entailed operating at very low values of variation of solids conversion (Δ*X* < 0.1). Under these conditions, the reactivity of fresh and used particles at the end of test 2 was practically identical. This situation was different in test 6 since the reactivity of the oxygen carrier had dropped substantially even operating at very low conversion values. Therefore, the loss of oxygen uncoupling capacity, together with this considerable reduction of reactivity, can explain the decrease in the combustion efficiency, η_c_, during tests with sulfur addition. It should be noted that operating at high oxygen carrier-to-fuel ratios with CaMnO_3_-based perovskites implies that the oxygen transference via oxygen uncoupling may be of higher relevance compared to the gas–solid reaction with lattice oxygen, which is required to obtain high η_c_ values in a continuous CLC unit.^24^

**Figure 7:**
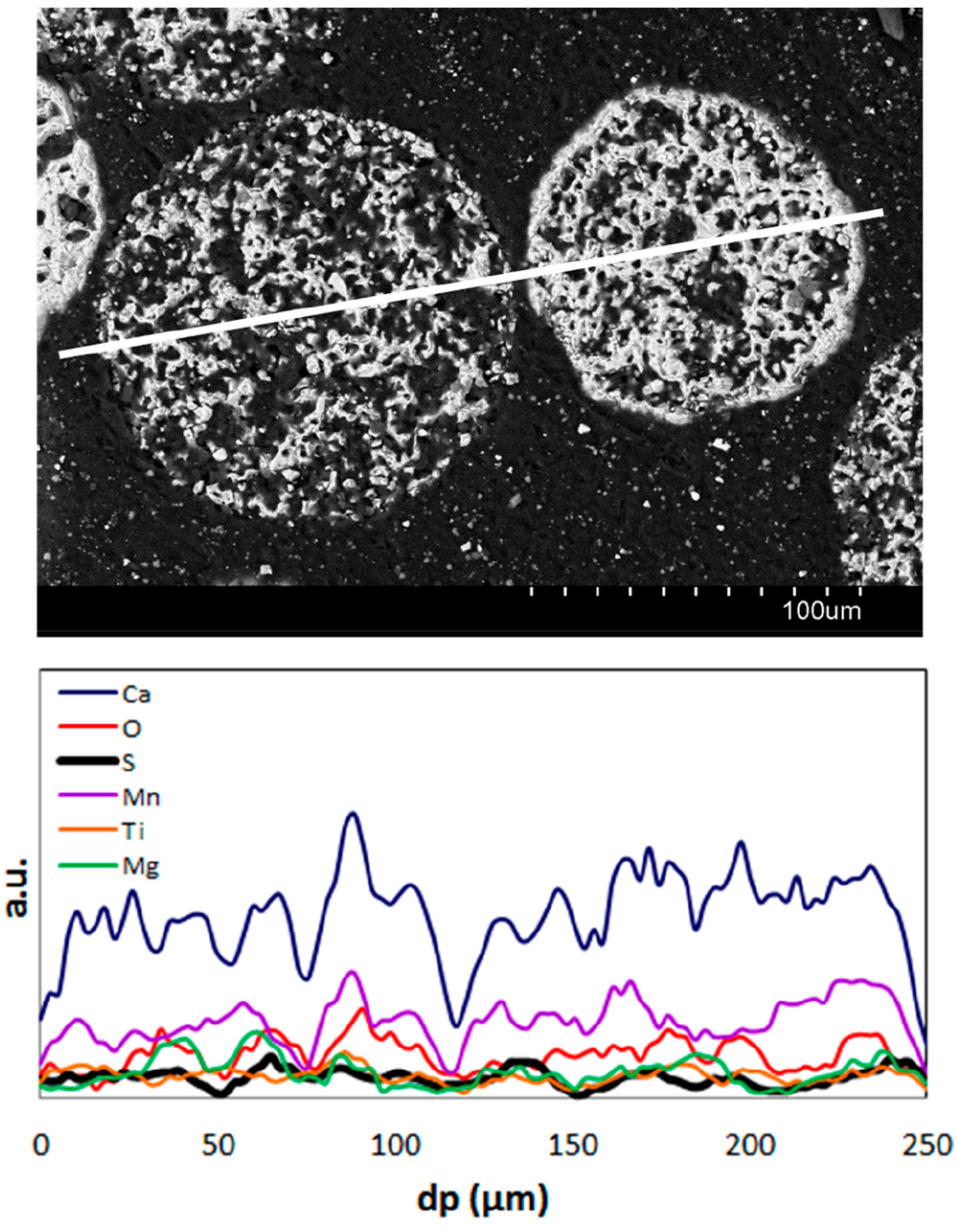
SEM micrographs and EDX line profiles of oxygen carrier particles extracted from the fuel reactor at the end of the experimental campaign with H_2_S addition.

The possible presence of sulfur in the oxygen carrier particles was analyzed by XRD, SEM-EDX, and ultimate analysis techniques. Table 1 shows that the CaSO_4_ crystalline phase was detected in the used particles extracted from the air reactor. This sulfur-based crystalline phase was also detected in the samples taken from the fuel reactor. Figure 7 shows the EDX line profiles of Ca, Mn, Mg, Ti, and S in the cross section of a pair of used particles extracted from the fuel reactor after test 6. This technique revealed that sulfur content was quite homogeneous inside the particles. These results differ from the findings obtained by Pachler et al.^25^ with this same material in a 120 kW_th_ chemical looping combustion pilot unit since these authors concluded that the only S-based crystalline phase detected was MgS, which was not homogeneously distributed as it was only accumulated on the surface of the particles. Thermodynamic studies carried out with the HSC 6.1 software^33^ show that at usual operating temperatures in a CLC unit (973–1273 K) highly reducing atmospheres are necessary in the fuel reactor to find MgS as a stable sulfur phase. On the contrary, under more oxidizing atmospheres where fuel is mainly oxidized to CO_2_ and H_2_O, as occurs during this experimental campaign carried out at the ICB-CLC-g1 prototype, the main stable S-based compound is CaSO_4_.

In order to quantify the amount of sulfur, some samples were analyzed by ultimate analysis in a Thermo Flash 1112. Data shown in Table 3 confirmed the accumulation of sulfur throughout the time of operation in the perovskite particles, reaching weight percentages higher than 0.5 wt % in the particles extracted from the air reactor at the end of the sour gas combustion tests. This value is considerably lower than the one expected of 1.2 wt % considering the amount of sulfur fed to the fuel reactor in the form of H_2_S (18 g S; see Figure 4) and the solids inventory at the pilot plant (1.5 kg) and that no sulfur was detected at any time in the gaseous streams at the outlet of the fuel and air reactors. This difference may be due to calculation errors or inaccuracies during experiments, lack of homogeneity in reactors sampling, loss of sulfur during the sample management and storage, or accumulation of sulfur over time on reactors walls and pipes that are part of the CLC unit. Anyway, it is unequivocal the accumulation of sulfur on the oxygen carrier particles and its negative effect on reactivity.

**Table 3:** Sulfur Concentration in Samples Extracted from the CLC Unit at Different Times of Operation

Test/sampling reactor	Accumulated time with H_2_S (h)	wt % S
5a/AR	8	0.15
5a/FR	8	0.20
6/AR	29	0.55
6/FR	29	0.41

It is important to note that during the 50 h of operation at hot conditions in the CLC unit the oxygen carrier particles did not show any signs of agglomeration in spite of the H_2_S supply. In this regard, the CaMn_0.775_Ti_0.125_Mg_0.1_O_3−δ_ oxygen carrier exhibited a better performance in terms of agglomeration resistance than the CaMn_0.9_Mg_0.1_O_3−δ_ material since the particles of the latter were agglomerated with just half of the sulfur fed into the CLC prototype, causing an unstable circulation of solids that led to stopping the experimental campaign.^23^

**Figure 8:**
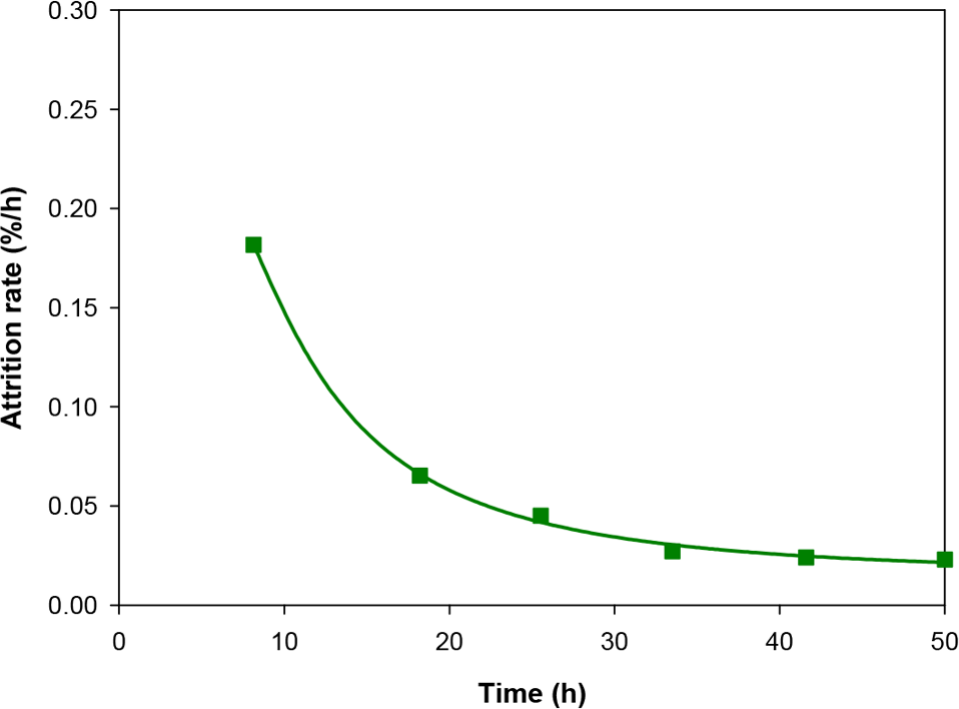
Attrition rate vs time of operation in the CLC prototype.

Finally, the mechanical behavior of the oxygen carrier particles was assessed by means of different parameters such as crushing strength, attrition resistance according to the air jet attrition index (AJI), particle lifetime, and mean particle size. Fresh and used particles exhibited a suitable and roughly constant crushing strength. The crushing strength of fresh particles was 1.6 ± 0.51 N, whereas the after-used particles (test 6) exhibited a similar value of 1.5 ± 0.42 N. Regarding the AJI parameter, fresh particles presented a value of 10.0%, whereas this parameter decreased up to 2.6% for used particles after 50 h of continuous operation in the CLC unit. The evolution of both parameters suggests an excellent stability of the oxygen carrier particles in a CLC unit.^34^Figure 8 illustrates the evolution of the attrition rate of CaMn_0.775_Ti_0.125_Mg_0.1_O_3−δ_ particles with time in the continuous CLC unit. The generation of fines during the first 10 h of operation was appreciable. However, the attrition rate rapidly stabilized at 0.025%/h, which entailed an estimated particle lifetime of 4000 h. The good performance of this material to abrasive attrition in the fluidized bed reactors was also corroborated by a minimum reduction in the average particle size evolving from 170 μm in the fresh particles to 160 μm in the used particles at the end of the experimental campaign; see data in Table 1.

Based on the above results, it could be concluded that the perovskite exhibited a good performance in terms of mechanical resistance throughout the experimental campaign with the presence of sulfur. In this regard, it is believed that the addition of H_2_S in the inlet fuel stream could be the beneficial factor that improved the mechanical behavior of the material due to the interaction of sulfur with the perovskite structure generating harder sulfur species such as CaSO_4_.^35^ However, the characterization of the CaMn_0.775_Ti_0.125_Mg_0.1_O_2.9−δ_ oxygen carrier also confirmed that the formation of CaSO_4_ had a poisonous effect, reducing its reactivity, combustion efficiency, and oxygen uncoupling capacity.

### 3.3 Regeneration of the CaMn_0.775_Ti_0.125_Mg_0.1_O_2.9−δ_ Oxygen Carrier

#### 3.3.1 Experiments in the Batch Fluidized Bed Reactor

The formation of CaSO_4_ disturbed the perovskite structure and clearly decreased its oxygen uncoupling capability. However, perovskite would be regenerated if CaSO_4_ could be converted into calcium oxide (CaO). Two different techniques have been analyzed in this work to try to remove the amount of sulfur accumulated in the CaMn_0.775_Ti_0.125_Mg_0.1_O_2.9−δ_ particles in the form of CaSO_4_ during the sour gas combustion tests performed in the CLC unit. The processes proposed to regenerate the material are the following:

##### 3.3.1.1 High-Temperature Redox Cycles

A direct reduction with H_2_ at high temperature may cause the releasing of the accumulated sulfur in form of SO_2_, see reaction R.6
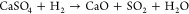
R.6

This reaction must be carried out at temperatures higher than 1173 K in order to enhance its selectivity to CaO. For lower temperatures, the dominant product after CaSO_4_ reduction is CaS according to reaction R.7.^36^
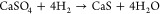
R.7

**Figure 9:**
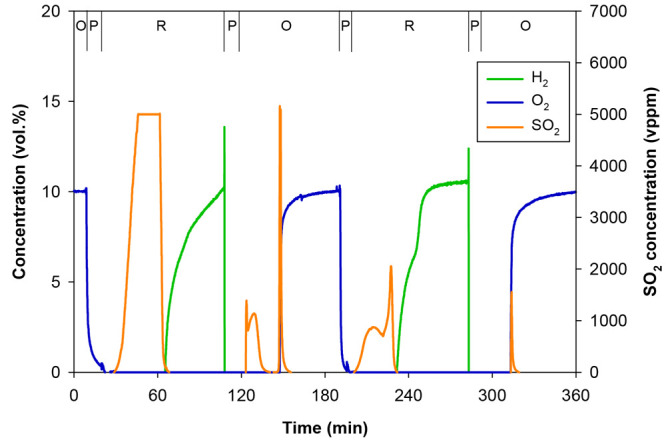
Gas product distribution during the cycles performed in the batch fluidized bed reactor to regenerate the CaMn_0.775_Ti_0.125_Mg_0.1_O_2.9−δ_ oxygen carrier at high temperature. *T* = 1273 K. Reducing agent = 10 vol % H_2_ (N_2_ to balance). O, oxidation; P, purge; R, reduction.

Figure 9 shows the gas product distribution during the redox cycles performed in the batch-fluidized bed at a high temperature (1273 K) using H_2_ as the reducing gas. At the beginning of the experiment, a mixture of air and nitrogen (10 vol % of O_2_; 90 vol % of N_2_) was fed to guarantee that all the oxygen carrier particles previously extracted from the CLC plant were completely oxidized. During this initial oxidation period, no sulfur compounds were detected since, as previously corroborated by different characterization techniques, all sulfur accumulated in the perovskite particles at the end of the sour gas combustion tests was already oxidized in the form of CaSO_4_.

During the first purge, the O_2_ concentration gradually decreased up to a value slightly below 1 vol %, which corresponded to the oxygen released by the perovskite through the oxygen uncoupling process. Sulfur started to be released in the form of SO_2_ shortly after the first reduction cycle began. During the period of time in which SO_2_ was released, the presence of H_2_ was not detected. This behavior suggests that the reduction reactions between the perovskite particles and H_2_ (reaction R.8) and between CaSO_4_ and H_2_ (reaction R.6) take place simultaneously.
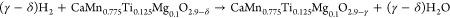
R.8

Once reduction was completed, any sulfur still remaining in the oxygen carrier should be in the form of CaS. During the next oxidation period, with a mixture of air and nitrogen (10 vol % O_2_; 90 vol % N_2_), CaS may be oxidized to CaSO_4_, see reaction R.9. In this case, two SO_2_ peaks were detected. Reaction R.10 could cause the release of SO_2_ during the oxidation period. However, Shen et al.^37^ concluded that this reaction has very little effect on the release of sulfur during the CaS oxidation process.
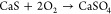
R.9
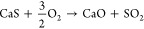
R.10

As can be observed from Figure 9, sulfur is released when reduction and oxidation semicycles are in progress, that is, when appreciable quantities of CaSO_4_ and CaS coexist. Once reduction and oxidation stages are completed, i.e., H_2_ and O_2_ start to be detected at the outlet of the reactors, SO_2_ release is interrupted as one of the two species becomes a minority compound. Therefore, the release of sulfur as SO_2_ may also be due to the solid–solid reaction between CaSO_4_ and CaS (reaction R.11).
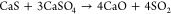
R.11

The amount of SO_2_ released during this first reduction cycle was significant since SO_2_ concentrations higher than 5000 vppm, the maximum value measured by the analyzer, were detected for more than 15 min. During the second redox cycle, the SO_2_ concentration values measured during the reduction and oxidation stages were lower than those obtained for the previous cycle, likely due to the lower amount of sulfur present in the material, which, in addition, would be more difficult to be regenerated.

##### 3.3.1.2 Low-Temperature Reaction with Mixtures of CO_2_ and H_2_O

In this case, regeneration is performed with the reduced solids extracted from the fuel reactor. To guarantee high reduction of the solids, the particles were first reduced to CaS according to reaction R.7 at 1073 K. At this temperature, CaSO_4_ is completely converted to CaS at a high reaction rate, but avoiding sulfur release as happening during the high-temperature regeneration process previously discussed. In a second stage, CaS reacts with CO_2_ and H_2_O to generate CaCO_3_ being the sulfur released in the form of H_2_S according to reaction R.12.
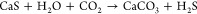
R.12

The equilibrium constant of this reaction is expressed as follows:^31^
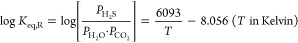
E.3

This reaction is favored at low temperatures (773–973 K) and high partial pressures of CO_2_ and H_2_O. This process was initially proposed to regenerate calcium sorbents in desulfurization processes for hot coal gasification gases obtaining at the same time a flue gas with a sufficiently high enough H_2_S concentration to be able to recover sulfur in a subsequent process such as the Claus process.^38^

**Figure 10:**
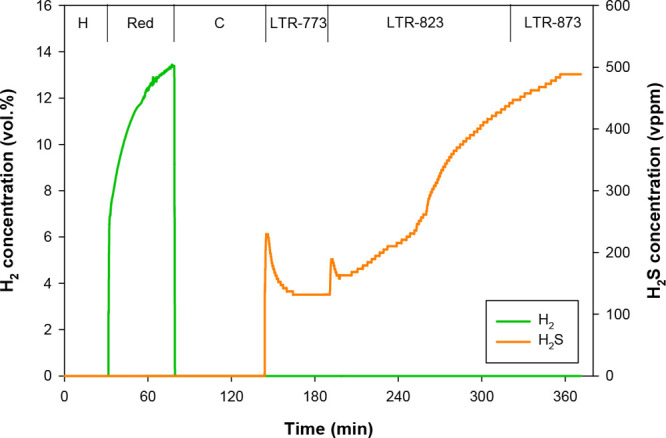
Gas product distribution during the test performed in the batch fluidized bed reactor to regenerate the CaMn_0.775_Ti_0.125_Mg_0.1_O_3−δ_ oxygen carrier at low temperature. Reducing agent = 15 vol % H_2_ (N_2_ to balance). Regeneration agent: 50 vol % H_2_O + 50 vol % CO_2_. H, heating (from room temperature to 1073 K); Red, reduction; C, cooling in N_2_ atmosphere (from 1073 to 773 K). LTR-773, LTR-823, and LTR-873: low temperature regeneration at 773, 823, and 873 K, respectively.

Figure 10 shows the gas product distribution during the tests performed in the batch-fluidized bed at low temperature (773–873 K) using a mixture of 50 vol % CO_2_ and 50 vol % H_2_O as regeneration agent. In this case, the batch of solids consisted of 75 g of used perovskite particles and 175 g of sand particles. The first stage of the experiment consisted of heating the reactor in a N_2_ atmosphere up to 1073 K, the temperature at which the reduction of the oxygen carrier with H_2_ (15 vol % H_2_ + 85 vol % N_2_) was conducted. As expected, during this reduction step, the presence of SO_2_ was not detected at the reactor outlet, since at 1073 K, reaction R.7 prevails over reaction R.6. Once the oxygen carrier particles were completely reduced, the temperature was lowered to 773 K in a pure N_2_ atmosphere. Next, a mixture of CO_2_ and H_2_O was fed into the reactor to start material regeneration. Likewise, a 200 L_N_/h air stream was fed into the postcombustor to oxidize the H_2_S formed by means of reaction R.12 to SO_2_. Thus, immediately after feeding the mixture of CO_2_ and H_2_O, SO_2_ began to be detected, which comes from H_2_S oxidation in the postcombustor. Figure 10 shows the corrected H_2_S concentration at the reactor exit. Initially, a small peak at 230 vppm was detected. Afterward, the H_2_S concentration decreased until it stabilized at a value around 140 vppm. As the H_2_S concentration at 773 K was not very high, the operating temperature was increased by 50 K. During the experiment carried out at 823 K, a progressive increase in the H_2_S concentration was observed, which indicated that an increase in temperature improved the rate of reaction R.12 and, consequently, the regeneration of the perovskite. After 2 h of operation at 823 K, the H_2_S concentration reached a value of 420 vppm. A subsequent increase of temperature up to 873 K did not lead to a considerable improvement in terms of perovskite regeneration, since after 45 min of operation at this temperature the H_2_S concentration stabilized at around 490 vppm.

#### 3.3.2 Characterization Results

The C28 perovskite samples subjected to regeneration tests were characterized by different techniques to evaluate whether the proposed sulfur removal processes were capable of increasing the reactivity and oxygen uncoupling capability of the material and reducing the amount of sulfur present in the particles. Figure 5 shows the oxygen uncoupling performance of the oxygen carrier particles after the regeneration tests carried out at high and low temperatures. As observed, the regeneration processes did not improve the oxygen uncoupling capability of the particles extracted from the CLC plant at the end of the experimental campaign with the addition of H_2_S to the fuel (test 6). Nevertheless, the regeneration processes did considerably increase the reactivity of the perovskite with CH_4_, see Figure 6, obtaining reduction conversion values similar to those exhibited by the perovskite after only 2 h of experimentation with H_2_S (test 2).

The regeneration of the oxygen carrier was also analyzed by determining the amount of sulfur in the particles by using ultimate analysis. The sulfur concentrations in the C28 material after conducting the regeneration tests at high and low temperatures were 0.20 and 0.16 wt %, respectively, values well below the ones obtained at the end of the experimental campaign with H_2_S addition, see Table 3. These values could have been even lower, that is, close to complete regeneration conditions, if more redox cycles had been performed at high temperature (Figure 9) or if the time of H_2_S formation with mixtures of CO_2_ and H_2_O at low temperatures had been extended (Figure 10). The reduction of sulfur accumulated in the particles after the regeneration processes was also revealed by XRD since the crystalline CaSO_4_ phase was not detected by this characterization technique.

From the characterization results of the regenerated samples, it can be concluded that there would be a tolerable sulfur content in the C28 perovskite particles to be used in a CLC plant for sour gas combustion. The upper limit could be estimated at 0.2 wt % S, a value at which it has been demonstrated that perovskite particles do not deactivate since their reactivity with CH_4_ is not affected.

#### 3.3.3 Implementation of Regeneration Processes in an Industrial CLC Unit

**Figure 11:**
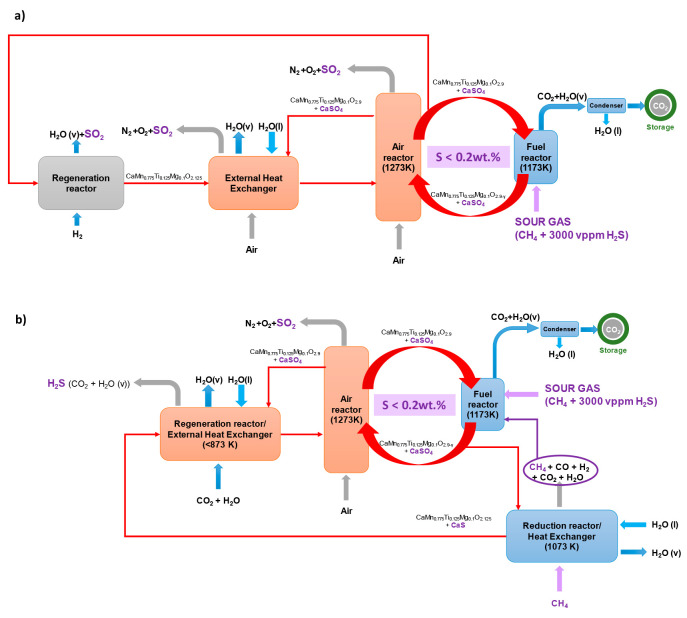
Integration of the regeneration process of the CaMn_0.775_Ti_0.125_Mg_0.1_O_3−δ_ perovskite at (a) high and (b) low temperatures in a CLC unit for the combustion of sour gas.

This section discusses the implementation of the previously described CaMn_0.775_Ti_0.125_Mg_0.1_O_2.9−δ_ perovskite regeneration stages in a commercial CLC facility using a sour gas stream as fuel. Figure 11 shows a scheme of a CLC unit including the high- and low-temperature sulfur removal steps. In both cases, it is considered that the operating temperatures of the fuel and air reactors are 1173 and 1273 K, respectively. The concentration of H_2_S in the gas stream fed to the fuel reactor is 3000 vppm, and the maximum quantity of sulfur allowed in the particles is 0.2 wt %. To control the amount of sulfur present in the material, part of the solid flow that leaves the air reactor or the fuel reactor is sent to the regeneration unit. These schemes also included adjacent fluidized beds acting as external heat exchangers to control the reactor temperatures. Note that a fraction of the produced heat in a CLC process should be extracted from hot solids in the reactors.

##### 3.3.3.1 High-Temperature Regeneration Process

In the high-temperature regeneration process, sulfur could be released from the oxygen carrier as SO_2_ if the air reactor operated at a temperature as high as 1273 K. This fact was confirmed during the oxidation semicycles performed in the batch fluidized bed reactor at 1273 K, see Figure 9. This hypothesis should be corroborated experimentally in a continuous CLC plant since the maximum temperature that could be reached in the ICB-CLC-g1 facility was 1223 K, and at that temperature, the presence of SO_2_ was never detected at the outlet of the air reactor. In this regard, the air reactor temperature in a 120 kW_th_ CLC unit could be likely somewhat higher than 1223 K, and at this temperature, SO_2_ was released in the air reactor.^25^ However, only a small fraction of total sulfur was released, and the remaining amount was accumulated in the solids. Note that the presence of SO_2_ in the gas stream at the outlet of the air reactor demands additional measures to avoid its emission to the atmosphere.

To allow regeneration of the perovskite, Figure 11(a) shows a proposed scheme where a diverted fraction of solids from the air reactor is regenerated. Thus, this solids stream is first introduced into the regeneration reactor where a H_2_ stream is fed to completely reduce the perovskite and decompose CaSO_4_ into CaO and SO_2_ according to reaction R.6. The reactions between H_2_ and the manganese oxide phases present in the perovskite structure are exothermic,^3^ which means that the regeneration process can be carried out at the temperature of 1273 K or even slightly higher. Rigorous mass and enthalpy balances would be required to determine the exact temperature of the regeneration reactor. The stream of regenerated oxygen carrier particles is then directed to the external heat exchanger prior to being fed into the air reactor.

##### 3.3.3.2 Low-Temperature Reaction with Mixtures of CO_2_ and H_2_O

Figure 11(b) shows the integration of the regeneration process of the CaMn_0.775_Ti_0.125_Mg_0.1_O_3−δ_ perovskite at a low temperature. In this case, sulfur should be as CaS. Therefore, the oxygen carrier should be previously reduced to form CaS and then be regenerated. Considering the high oxygen carrier-to-fuel (ϕ) values required for this oxygen carrier, it was determined that the oxidation degree was similar both in the fuel and air reactors, and sulfur was present in both reactors as CaSO_4_. Initially, a diverted fraction of solids from the fuel reactor is reduced with a CH_4_ stream at 1073 K. The reactions between CH_4_ and the C28 perovskite are endothermic, and the stream of unburned gases (CH_4_, CO, and H_2_) is recycled to the fuel reactor for complete conversion. Subsequently, the temperature of the solids should be decreased to 873 K to be regenerated by a CO_2_/H_2_O mixture, as described in Section 3.3.1. Thus, the external heat exchanger of the CLC unit may be used as the regenerator, where a stream of CO_2_ and steam is fed to transform CaS into H_2_S according to reaction R.12. Thus, a gas stream with a high concentration of H_2_S is obtained at the outlet of this reactor, which can be subsequently converted to byproduct elemental sulfur in a Claus process or alternatively converted to valuable H_2_SO_4_ in a wet gas sulfuric acid (WSA) process unit.

Rigorous mass and enthalpy balances as well as a dedicated techno-economic assessment would be required to evaluate the potential of the proposed options to regenerate the perovskite material. Each option has some advantages over the other. In this regard, the high-temperature option may produce a highly concentrated SO_2_ stream ready to be used. However, it may require the use of a high flow of hydrogen to reduce the oxygen carrier particles in the regenerator reactor. Note that hydrogen is preferred in order to maintain a high temperature in the regeneration reactor. In the low-temperature option, only an additional reducer would be required, as the heat exchanger might be used as the low-temperature regenerator. Likewise, this option would require recirculating part of the CO_2_ and steam streams produced at the outlet of the fuel reactor to feed them into the regeneration reactor.

**Figure 12:**
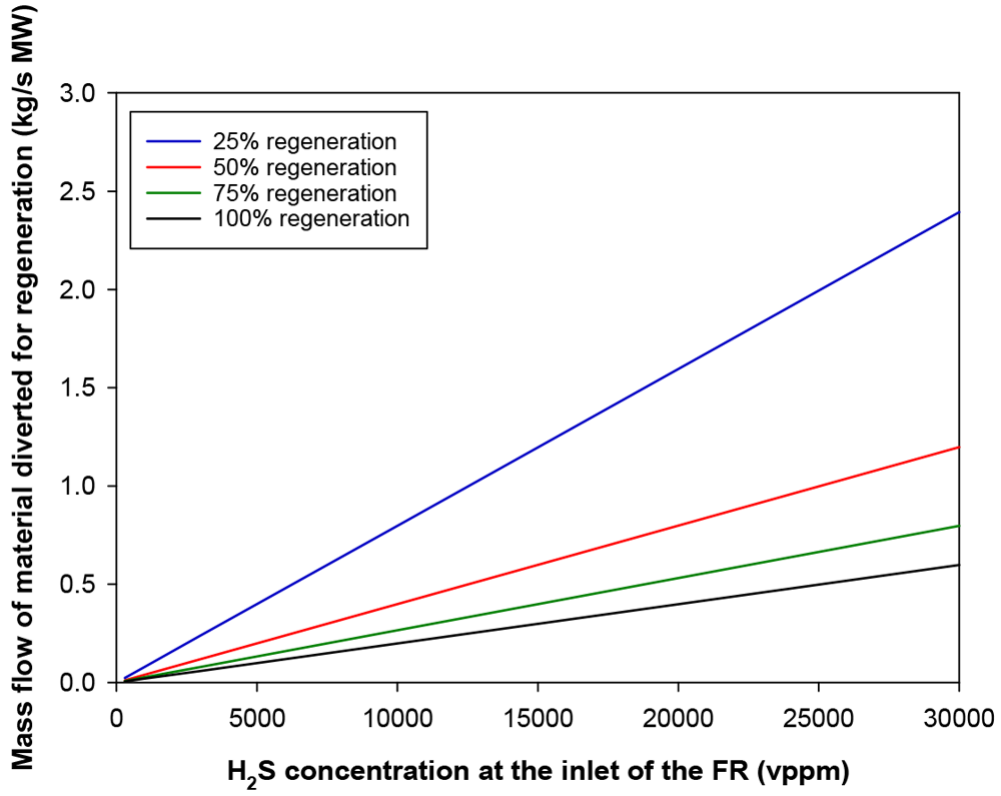
Effect of the H_2_S concentration at the inlet of the fuel reactor on the mass flow of oxygen carrier particles diverted to the regeneration unit for different percentages of regeneration. Maximum amount of sulfur accumulated in the oxygen carrier particles = 0.2 wt %. Power = 1 MW_th_ of sour gas.

In a next step, we calculated the percentage of the solids stream leaving the air reactor or fuel reactor that should be diverted to the regeneration stage of the industrial CLC facility in order to maintain the amount of sulfur accumulated in the particles low enough so that the perovskite exhibits a suitable behavior for the combustion of sour gas. As mentioned above, this amount was set at 0.2 wt % to minimize the detrimental effect of sulfur accumulation on the oxygen carrier particles. The calculations were done per MW of thermal power fed to the fuel reactor as sour gas. Figure 12 shows the variation of the mass flow of material diverted to the regeneration stage as a function of the H_2_S concentration in the sour gas and the regeneration degree. Thus, for a sour gas stream with a H_2_S concentration of 3000 vppm, the mass flow of material diverted to the regeneration stage is 0.06 kg/s MW when 100% of the sulfur contained in the diverted particles is regenerated. The higher the H_2_S concentration or the lower the regeneration degree is, the higher the mass flow of the oxygen carrier that is diverted to the regeneration unit.

In summary, the amount of sulfur present as H_2_S in the sour gas may have a very significant effect both from an operational and an economic point of view when the CaMn_0.775_Ti_0.125_Mg_0.1_O_2.9−δ_ perovskite is considered as an oxygen carrier. In this regard, depending on the H_2_S concentration in the sour gas, alternatives to the in situ regeneration of the sulfated perovskite can be considered such as pretreating the fuel gas coming into the CLC plant in a sweetening unit or installing an oxygen polishing unit at the outlet of the fuel reactor to convert the unburned compounds coming from the sour gas stream (CO, H_2_, and CH_4_), which were generated due to the gradual deactivation of the oxygen carrier, into CO_2_ and H_2_O. Therefore, it is proposed as future work to carry out a thorough techno-economic analysis that evaluates all these options if it is intended to scale up the use of this material in an industrial CLC process fed with natural gas.

## 4 Conclusions

The performance of a CaMn_0.775_Ti_0.125_Mg_0.1_O_2.9−δ_ perovskite as oxygen carrier to burn sour gas with H_2_S concentrations up to 3000 vppm has been examined during 29 h of combustion in a 500 W_th_ CLC prototype. All the sulfur present in the fuel gas was accumulated in the form of CaSO_4_ in the oxygen carrier particles, which were deactivated in terms of an important decrease in the reactivity, oxygen uncoupling capability, and combustion efficiency. These effects depended on the mass fraction of sulfur accumulated in the perovskite.

In order to regenerate the oxygen carrier, two different methods were proposed and evaluated: (1) direct reduction of CaSO_4_ with H_2_ at high temperature (1273 K) and (2) formation of H_2_S with mixtures of CO_2_ and H_2_O at low temperature (<873 K). The application of both methods led to the regeneration of the material with a considerable reduction of the sulfur content in the oxygen carrier particles and an increase in the reaction rate with CH_4_ compared to that exhibited by the used particles at the end of the experimental campaign with the addition of H_2_S. In this respect, a tolerable limit of sulfur concentration in the CaMn_0.775_Ti_0.125_Mg_0.1_O_2.9-δ_ perovskite particles has been set at 0.2 wt % in order to prevent their deactivation.
